# Nonsurgical Management of a Giant Coronary Artery Aneurysm

**DOI:** 10.7759/cureus.1478

**Published:** 2017-07-16

**Authors:** Priscilla Wessly, Shahabuddin Soherwardi, Norman Allen

**Affiliations:** 1 Department of Internal Medicine, Howard University Hospital; 2 Cardiovascular Diseases, Providence Hospital

**Keywords:** coronary artery aneurysm, giant aneurysm, coronary angiography

## Abstract

Coronary artery aneurysms (CAAs) are rare dilations of arterial segments. These aneurysms are mostly caused by atherosclerosis. Due to the rarity of this condition, there are no official guidelines for its management; therefore, management is mainly based on case reports. We present a patient with a giant CAA in the left anterior descending artery who was treated medically. At 12-month follow-up,  he was asymptomatic and had no complications.

## Introduction

Coronary artery aneurysm (CAA) has an incidence of up to 4.9% and is usually detected incidentally during coronary angiography. Giant CAA is even more rarer with a reported incidence of 0.02%. These aneurysms are mostly caused by atherosclerosis. They can be asymptomatic or present with potential lethal presentation ranging from angina, myocardial infarction (MI), tamponade, and death.  There is no general consensus on the management of CAA. Given its increasing incidence, it is important to know about its diagnosis, associated complications, and various forms of management. Herein, we present a patient who was found to have a giant CAA in the left anterior descending (LAD) artery and was treated medically. He remained asymptomatic with no complications at one year follow up. 

## Case presentation

A 72-year-old male with past medical history of hypertension and chronic hepatitis C treated with ledipasvir and sofosbuvir presented to our clinic with a history of three months of lightheadedness. The patient described the lightheadedness as gradual in onset, aggravated by exertion, and relieved by rest. He denied chest pain, syncope, palpitation, shortness of breath, or history of febrile illness in childhood. Physical examination including vitals was unremarkable.

**Figure 1:**
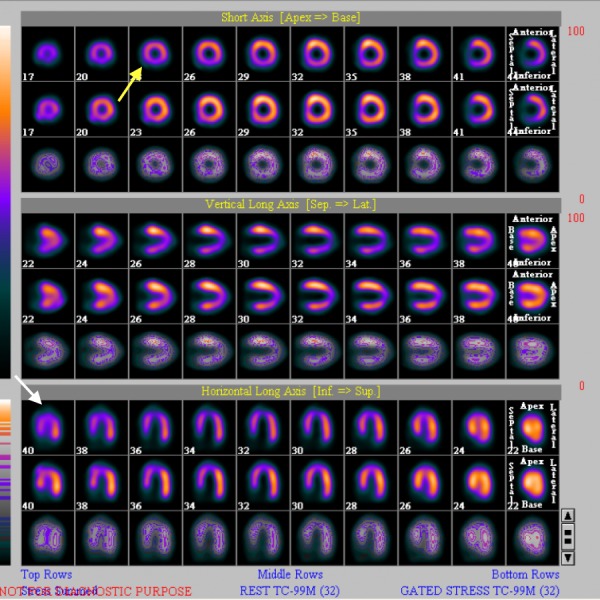
Nuclear stress test showing small area of ischemia in the inferior-septal left ventricular wall (white arrow) and a moderate size area of ischemia in the inferior left ventricular wall (yellow arrow).

Electrocardiogram (ECG) showed non-specific ST-T changes. Echocardiography showed left ventricular ejection fraction of 55% with no regional wall motion abnormalities. A nuclear stress study revealed a small area of ischemia in the inferior-septal left ventricular wall and a moderate size area of ischemia in the inferior left ventricular wall (Figure 1).

**Figure 2:**
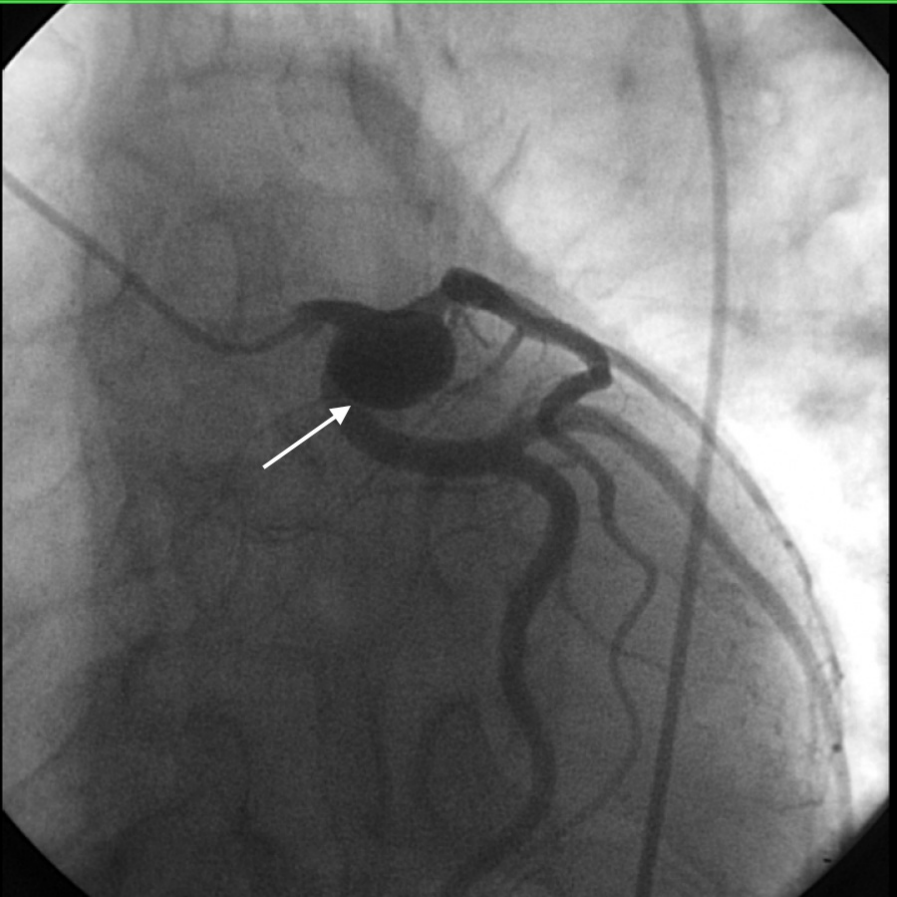
Coronary angiogram showing aneurysm in the left anterior descending (LAD) coronary artery (arrow).

A subsequent coronary angiogram showed a large aneurysm in the proximal part of LAD coronary artery (Figure 2). Except for 50% stenosis of LAD coronary artery, no other stenosis was seen.

**Figure 3:**
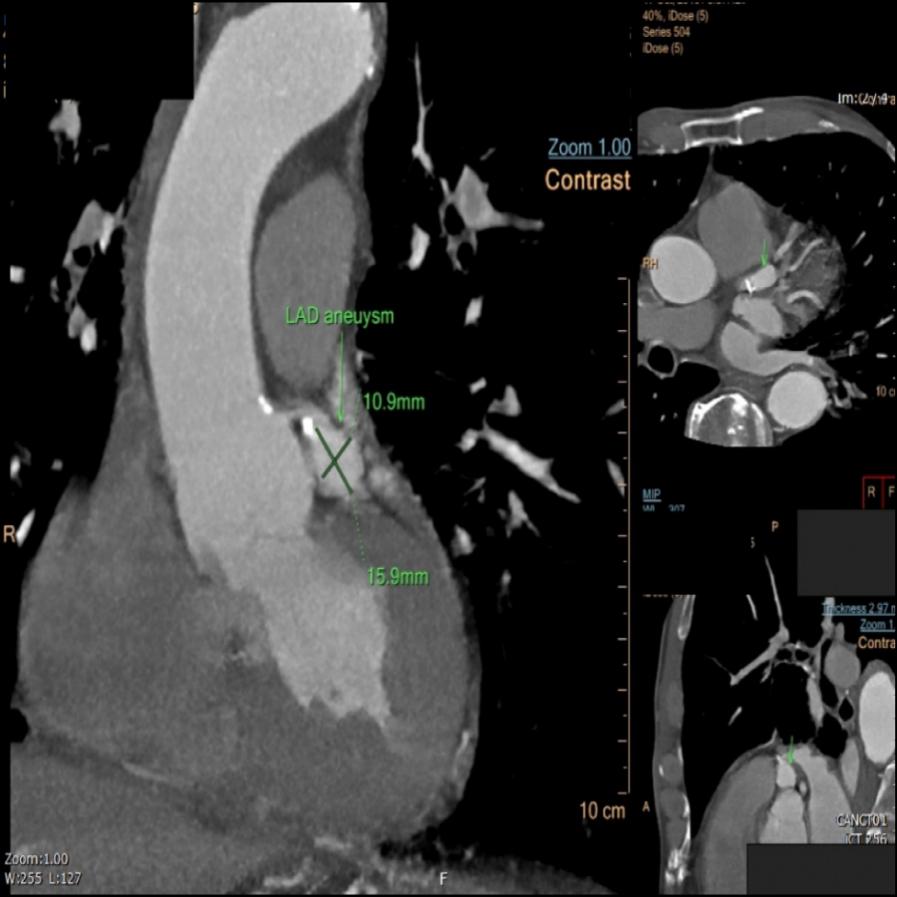
Computed tomography angiogram heart coronary showing aneurysm in the LAD coronary artery (green arrow).

The patient declined surgical intervention and therefore medical management was chosen. He was started on aspirin along with anticoagulation with warfarin.  A cardiac computed tomography (CT) angiogram done at follow up showed a stable aneurysm measuring about 16 mm by 11 mm with coronary calcium score of 186 (Figure 3). Post-initiation of medical therapy, at 12 months follow up, the patient has been symptom-free and without any complications. 

## Discussion

CAA was first described in a post-mortem study in 1761 by Morgagni [1 ]. However, the first CAA in a living patient was diagnosed via coronary angiography by Munkner et al. in 1958 [2]. CAA has been defined as coronary dilatation that exceeds the diameter of the adjacent normal segments or diameter of patient’s largest coronary vessel by 1.5 times [3]. They are uncommon but have been diagnosed with increasing frequency due to the advent of new technology. Incidence varies from 0.3% to 4.9% [4]. CAAs are called giant if their diameter exceeds the reference coronary artery diameter by >4 times or if they are >8 mm in diameter [5].

The most frequent site of coronary aneurysm is the right coronary artery (RCA), followed by the circumflex, LAD, and the left main coronary artery. Topaz et al. studied coronary angiography in 13,010 adults and CAA was found in RCA in 62%, circumflex in 27%, LAD in 11%, and left main in 0.1% [6].

The most common etiologic factor is atherosclerosis (50%) and the next common cause is congenital (20%-30%). Other causes are Takayasu’s arteritis, lupus, rheumatoid arthritis, Marfan syndrome, and Ehler danlos syndrome. CAAs are also seen with infection, drug use, trauma, or postpercutaneous intervention (PCI). In an autopsy review done by Daoud et al., 52% were atherosclerotic, 17% congenital, 11% mycotic-embolic, 11% dissecting, and 4% syphilitic [7]. Our patient did not have any history of connective tissue disease, vasculitis, trauma, or infectious process; the most likely cause is atherosclerosis in light of underlying non obstructive coronary artery disease in the LAD.

In majority of cases, CAAs are asymptomatic and can present at any age; those related to atherosclerosis usually present in later life than congenital cause.

CAAs, being dilated, are subject to spasm, thrombosis, dissection, and spontaneous rupture and can be a potential cause for MI [8-9]. Therefore, it is important to treat them. Since CAA is rare there is no standard management guidelines. Treatment options include surgical, PCI, or medical approach. The appropriate treatment is controversial and depends on the particular clinical situation. There are both surgical and nonsurgical management options which must be discussed broadly with the patient.

Based on case reports, the decision to surgically manage CAAs was based on the severity of coexisting coronary stenosis. Surgical management was usually done in symptomatic patients with obstructive coronary artery disease or evidence of emboli causing myocardial ischemia. PCI involves using polytetrafluoroethylene-covered stents and is known for its ease of use and effectiveness [4]. The medically conservative therapy generally aims at preventing thromboembolic complications with the administration of antiplatelet and anticoagulation. Those on medical therapy are monitored very closely. The optimal time for follow up is not definitive but suggested time is three months [10].

## Conclusions

In this study, for our patient with a giant aneurysm in the LAD coronary artery, we have used a medical approach since our patient was hesitant about surgery. The patient has remained symptom-free and without complications at one-year follow-up. To the best of our knowledge, there is only one case report which showed good outcome with medical therapy. Coronary angiography is the gold standard for diagnosis. The optimal therapy is unknown and controversy persists regarding modality of management.  Anticoagulation is the mainstay for medical management but needs aggressive monitoring given complications.
